# New Insights in the Diagnosis and Treatment of Atrial Fibrillation in Patients with Hypertrophic Obstructive Cardiomyopathy

**DOI:** 10.3390/jcm15083014

**Published:** 2026-04-15

**Authors:** Cristina-Mihaela Angelescu, Toma Andrei Iosifescu, Cristina Ioana Căldăraru, Andrei Daniel Dermengiu, Oana Raluca Ioniţă, Horaţiu Moldovan, Lucian Florin Dorobanţu

**Affiliations:** 1 Faculty of Medicine, “Titu Maiorescu” University, Dâmbovnicului Street 22, 040441 Bucharest, Romania; tomaiosifescu@yahoo.com (T.A.I.); andrei.dermengiu@nord.ro (A.D.D.); ludorobantu@gmail.com (L.F.D.); 2 Provita Medical Group, Department of Cardiac Surgery, Nord Pipera Hospital, Dimitrie Pompeiu Street 9, 020335 Bucharest, Romania; dr.caldararu.cristina@gmail.com (C.I.C.); oana.savu@yahoo.com (O.R.I.); 3 Department of Cardiology, “St. Pantelimon” Emergency Clinical Hospital, Pantelimon Street 340-342, 021659 Bucharest, Romania; 4 Department of Cardiac Surgery, Clinical Emergency Hospital of Bucharest, Floreasca Street 8, 014461 Bucharest, Romania; horatiu.moldovan@umfcd.ro

**Keywords:** hypertrophic obstructive cardiomyopathy, atrial fibrillation, atrial myopathy, myosin inhibitor, septal reduction therapy, cardiac mitotropic agents

## Abstract

Hypertrophic cardiomyopathy (HCM) is the most common genetic disease in the general population, with a variable phenotypic expression and symptomatology. Atrial fibrillation (AF) is the most common arrhythmia identified among patients diagnosed with HCM. Treatment of both AF and HCM has continuously evolved over time, leading to a significant improvement in the prognosis and life expectancy of symptomatic patients. Numerous studies have demonstrated that the risk of developing this arrhythmia correlates with atrial morphological, functional and electrical remodeling, a process known as atrial myopathy. Once a first episode of AF is diagnosed, permanent anticoagulation is required among patients diagnosed with HCM, regardless of the CHA_2_ DS_2_-VA score. Additionally, atrial cardiomyopathy is associated with an increased thromboembolic risk, independent of AF presence, in patients with stable sinus rhythm, in the context of atrial mechanical and endothelial dysfunction. This article aims to evaluate the current scientific evidence and treatment approaches in patients diagnosed with HCM.

## 1. Introduction

HCM is a primary myocardial disorder, frequently familial, with a heterogeneous phenotypic expression, unique pathophysiology and variable clinical course, characterized by left ventricular (LV) hypertrophy, in the absence of associated conditions that could explain it, such as arterial hypertension, aortic stenosis, and athlete’s heart [1].

It is the most common genetic cardiovascular disease, with a prevalence of 1:200 [2]. Most cases have been reported in the United States, Canada, Western Europe, Israel, and Asia (Japan and China) [2]. Its prevalence exceeds the number of patients diagnosed in daily practice (estimated to be 1:100,000 population), suggesting that the majority of individuals remain undiagnosed, are often asymptomatic, and do not experience cardiovascular events during their lifetime [3].

Being an autosomal dominant disease, it should occur with equal frequency in both women and men. However, studies indicate a higher prevalence in men, suggesting that this pathology is underdiagnosed in women, who are often evaluated at more advanced stages of heart failure and at older ages [3].

## 2. Methodology

This manuscript is a narrative review which aims to provide the latest updates on the diagnosis and treatment of atrial fibrillation (AF) in patients diagnosed with hypertrophic obstructive cardiomyopathy (HOCM). Our option was a narrative approach, due to the heterogeneity of available studies, making a systematic review less suitable. This approach allows for qualitative synthesis of the current literature, highlighting the latest developments in this genetic pathology.

A literature search was conducted using PubMed, Web of Science and Google Scholar up to January 2026, using keywords such as: “hypertrophic obstructive cardiomyopathy”, “atrial fibrillation”, “catheter ablation”, “atrial myopathy”, “risk factors”, “septal reduction therapy”, and “cardiac mitotropic agents”.

No formal systematic selection process (PRISMA) was applied, given the narrative structure of this review. Studies were selected based on relevance, scientific quality and contribution to understanding the risk factors, pathophysiology and treatment options.

Data were analyzed qualitatively, focusing on findings, study limitations and sources of bias.

All images and patient data were collected from our institutional database and were anonymized prior to analysis.

This narrative manuscript provides an overview of this topic, highlighting current updates and future research directions.

## 3. HCM Genetics

The disease has a Mendelian, autosomal dominant inheritance pattern, with descendants having a 50% risk of inheriting this pathology.

HCM is caused by mutations in 11 genes that encode the light and heavy contractile myofilaments that are components of sarcomeres and Z-discs. The most important genes involved in the development of HCM are MYH7 and MYBPC3, affecting 70% of individuals diagnosed with this pathology. The genes encoding troponin T (TNNT2) and troponin I (TNNI3) account for less than 5% of cases. Studies have shown a more severe progression in patients with positive genetic tests compared to those with inconclusive results, particularly in patients with heavy chain mutations (MYH7 and MYBPC3). However, there is insufficient data to determine whether the presence of multiple sarcomeric gene mutations in the same patient is associated with earlier onset or increased severity. Genetic testing is particularly important for identifying patients with metabolic and storage diseases that mimic HCM but require different management.

There are cases in which the HCM phenotype does not fully manifest until adolescence, when accelerated growth is accompanied by increased ventricular wall thickness and an extensive cardiac hypertrophy distribution. Sometimes, structural changes become evident in adulthood or even old age, with individuals being frequently asymptomatic and free of arrhythmic events. Family members carrying the HCM genes, who do not develop left ventricular hypertrophy (LV), still exhibit clinical and imaging changes (diastolic dysfunction, myocardial crypts, elongated mitral valves, myocardial scars or ECG abnormalities) [3].

AF is the most common arrhythmia found among patients diagnosed with HCM, predisposing to cardiac chamber dilation, diastolic dysfunction, decreased cardiac output, increased risk of thromboembolic events, and being poorly tolerated, especially among patients with HOCM [4,5].

## 4. Risk Factors for AF in HOCM

Paroxysmal, persistent, or permanent AF occurs in 20–25% of patients with HCM, compared to 1–2% in the general population. Its incidence increases with age, the degree of left atrial (LA) dilation, and the severity of its dysfunction. Symptomatic paroxysmal AF negatively affects quality of life, with patients presenting to the emergency room for cardioversion. It is noteworthy that mortality from AF itself in patients with HCM is low, being <1% per year and mainly caused by ischemic vascular accidents in the absence of anticoagulation [3].

Sarcomeric mutations appear to be associated with a higher prevalence and earlier onset of AF compared with non-familial forms of HCM. Some studies show that patients diagnosed with MYH7 (the most frequent mutation) and MYBPC3 mutations have a high prevalence of low voltage areas on electroanatomical mapping, corresponding to large areas of scar and predisposing to AF.

Additionally, some studies suggest that mutations encoding thin myofilament genes are associated with a higher prevalence of diastolic dysfunction and heart failure symptoms, compared with genes coding thick chains. Furthermore, the missense variant Mt228Thr in the ACTN2 gene encoding α-actinin-2 has been associated with familial forms of apical and midventricular HCM, as well as with early onset of AF and atrioventricular blocks.

Although less studied, mutations in non-sarcomeric genes may increase the risk of AF. Therefore, Orenes-Piñero et al. identified the 344T > C polymorphism in the CYP11B2 gene as an independent predictor of AF in patients with HCM. This polymorphism has been associated with activation of the renin–angiotensin–aldosterone system, increased aldosterone levels and, consequently, atrial fibrosis and remodeling [4].

A study published in 2022 that included 949 patients suggests that the incidence of AF progressively increases following septal myectomy, reaching a maximum of 42.5% at 10 years postoperatively. This study identified the following risk factors for the occurrence of AF: preoperative LA dilation, pulmonary artery systolic pressure, at least moderate postoperative mitral regurgitation, increased interventricular septum thickness, age, and persistent postoperative dynamic obstruction [6].

It has been demonstrated that an indexed LA volume > 40 mL/m^2^ represents an important parameter associated with the risk of developing AF [7].

The incidence of AF after septal myectomy is significantly higher in patients with frequent extrasystolic supraventricular arrhythmia on Holter ECG. Frequent atrial extrasystoles can predict the onset of AF in patients with HOCM [8,9].

Multimodal assessment of LA function using cardiac magnetic resonance (CMR) and transthoracic echocardiography revealed the correlation between left atrio-ventricular coupling index and LA ejection fraction (LAEF) with the risk of developing AF. LAEF < 43% identifies patients with a 2- to 9-fold increased risk of AF, compared to other patients with HCM, requiring frequent monitoring with a 48 h Holter ECG or an implantable loop recorder [10]. Detection of increased extracellular fluid on CMR among patients who develop AF, compared with the control group (35.4% vs. 31.8%), suggests that extracellular fluid is a prognostic parameter for the AF onset [11].

Recent studies emphasize the importance of right ventricular (RV) dysfunction in the pathogenesis of AF in patients with HOCM. The RV peak filling rate, a parameter assessed using CMR software (CVI software, version 5.3.4, Circle Vascular Imaging; Siemens Skyra (3.0 T) magnetic resonance imaging system), which quantifies the diastolic filling rate of the RV, highlights that ventricular compliance is an independent predictor of new-onset AF. Atrio-ventricular uncoupling is associated with increased right atrial (RA) pressures, electrical remodeling and atrial stretch [12].

Age between 40 and 50 years has been identified as an independent risk factor for the development of AF, although individuals < 30 years have a much lower risk for developing arrhythmias [9].

Renal dysfunction is another factor that contributes to the occurrence of new-onset AF. The correlation between serum creatinine levels, reduced eGFR, diastolic dysfunction (increased indexed LA volume: 51.1 ± 23.2 vs. 32.4 ± 12.1 mL/m^2^, reduced a′ wave velocities at the mitral annulus, and increased end-diastolic and end-systolic LV volumes assessed by echocardiography) and AF has been demonstrated. The association of reduced eGFR and dilated LA significantly increases the risk of new-onset AF in patients with HCM [11].

Scientific data on AF risk factors in patients with HCM are heterogeneous, clinically limited, and largely based on observational studies. Imaging and electrophysiological studies suggest that atrial remodeling, as assessed by fibrosis severity, electrical heterogeneity, and atrial contractile dysfunction, is responsible for AF pathogenesis and recurrence in patients with HCM. However, the strength of evidence is limited by small studies, different methodologies, and the absence of standardized protocols. Studies that have evaluated biomarkers, such as galectin-3, atrial natriuretic peptides, and plasma proteomic profiling, have provided interesting data, but they are not yet validated and reproducible. It is noteworthy that most of the data in the literature have been extrapolated from non-HCM populations, and their validation among patients with HCM requires targeted, dedicated studies. The VANISH study, which is a randomized, controlled trial, delivers a range of supporting evidence in patients with HCM, but offers indirect information regarding the risk of developing atrial fibrillation. Large, prospective, randomized trials are needed to build predictive models, optimizing the management of these patients.

### HCM Phenocopies and AF

The phenotype characterized by LV hypertrophy is represented by sarcomeric HCM (the most common form, accounting for up to 60% of cases), but also by a series of phenocopies (nonsarcomeric HCM). HCM phenocopies are classified as follows:

1. Storage diseases (cardiac amyloidosis, Anderson–Fabry disease, glycogen storage disorders, mucopolysaccharidoses: Hurler syndrome, sphingolipidoses: Gaucher and Niemann–Pick diseases, and fatty and metabolic defects.
2. Other rare genetic diseases: mitochondrial disease (Mitochondrial Encephalopathy, Lactic Acidosis and Stroke-like episodes syndrome—MELAS, and Myoclonic Epilepsy with Ragged-Red Fibers syndrome—MERRF); rasopathies (Noonan Syndrome and Neurofibromatosis type 1); and neuromuscular diseases (Friedreich’s ataxia, Duchenne and Becker dystrophy, Myotonic dystrophy type 1, and Desminopathies). Accurate clinical findings and imaging are essential to differentiate phenocopies from sarcomeric HCM. Electrocardiogram, echocardiography, CMR, laboratory and genetic tests play particularly important roles in the definitive diagnosis. Diagnosis of phenocopies is very important for correct management and treatment, as targeted treatments exist for cardiac amyloidosis, Fabry disease, mitochondrial and metabolic diseases.

The prevalence and incidence of AF in these phenocopies differ from sarcomeric HCM due to distinct pathophysiological mechanisms determining myocardial infiltration, atrial remodeling and fibrosis. For example, cardiac amyloidosis predisposes to early-onset AF, being frequently associated with severe atrial dilatation, fibrosis and impaired atrial compliance. Anderson–Fabry disease is a lysosomal storage disorder involving concentric LV hypertrophy, but also asymmetric septal hypertrophy, with variable atrial involvement. Diagnosis of AF in phenocopies requires close monitoring of affected individuals, requiring anticoagulation with direct anticoagulants or acenocoumarol, in a similar manner to sarcomeric HCM (TERESI).

In a recent study on the prevalence of AF in patients diagnosed with ATTR amyloidosis, 48% of participants were diagnosed with this arrhythmia prior to enrollment in the study, and another 48% were subsequently diagnosed during the investigations. AF is a common arrhythmia among patients with cardiac amyloidosis, predisposing them to a high risk of ischemic stroke. Identification of this arrhythmia requires the initiation of anticoagulant treatment regardless of the CHA2DS2-VASC score.

In a review published in 2023 on the incidence and prevalence of AF among patients with Fabry disease, an average of 12.2% of patients affected with AF is reported, with the majority of individuals being asymptomatic.

Among patients diagnosed with the PRKAG2 mutation, a study published in 2020 reports a percentage of 18% of patients with AF at the beginning of the study. In a median follow-up period of 6 years, 29% of participants were diagnosed with this arrhythmia. A high percentage (32%) of patients developed this arrhythmia at a young age (before 35 years), and 71% of individuals also had associated LV hypertrophy at echocardiography evaluation.

In patients known to have myotonic dystrophy type 1, the average prevalence of AF was 10.9% (70 times higher than in the general population), with risk factors associated with this arrhythmia being male sex, conduction disorders detected on electrocardiogram and echocardiographic prolonged electromechanical delay [13,14,15,16].

## 5. Diagnosis and Evolution of Atrial Myopathy in Patients with HCM

### 5.1. The Relationship Between Left Atrial Myopathy and AF

Atrial myopathy is defined as the structural and functional remodeling of the LA, accompanied by atrial dilation and predisposition to atrial arrhythmias, particularly AF.

Along with the hemodynamic factors involved in the occurrence of atrial myopathy (such as left ventricular (LV) remodeling, diastolic dysfunction, mitral regurgitation, and dynamic obstruction in the LV outflow tract (LVOT)), we also mention the modifiable risk factors (obesity, diabetes, hypertension, and NYHA functional class), but also non-modifiable risk factors (age and female gender), all of which contribute to increasing AF risk [17].

Furthermore, a number of echocardiographic parameters, including LA volume > 40 mL/m^2^, atrio-ventricular coupling index and LA function, predict the onset of AF.

The atrio-ventricular coupling index (defined as the ratio of LA end-diastolic volume to LV end-diastolic volume) is independently associated with the risk of AF, suggesting that early changes in LV compliance and intracardiac pressures could predict impaired atrial function before morphological changes occur [17].

In addition, an LA strain < 23.4% is an independent risk predictor of developing AF, regardless of LA diameter [18].

In a cohort of 208 patients, it was shown that the total atrial conduction time, measured by tissue Doppler, was associated with new-onset AF, independent of LA volume [19].

Among the biomarkers associated with an increased incidence of AF or frequent recurrence post-ablation, studies mention high-sensitive troponin T (not troponin I), which has been significantly increased in patients with recurrent paroxysmal AF compared to those without recurrence (11.7 ± 8.3 pg/mL vs. 8.4 ± 4.7 pg/mL) [20]. NT-pro-BNP is a good marker of atrial remodeling in patients with HOCM and predicts the risk of AF recurrence after transcatheter ablation. In a prospective study on AF ablation, NT-proBNP levels ≥ 291 pg/mL in paroxysmal AF and ≥368 pg/mL in persistent AF demonstrated high sensitivity and specificity in identifying patients at a high risk of AF recurrence over a 12-month follow-up [21].

Inflammatory markers, particularly C-reactive protein (CRP), have been useful in identifying patients at risk of developing AF, correlating with the degree of fibrosis and atrial remodeling. Patients with elevated CRP levels (>2.7 mg/L) had a 60% higher risk of AF recurrence compared to patients with normal levels (<0.8 mg/L) [20].

Patients with persistent AF have elevated plasma galectin-3 levels, a biomarker which is an independent predictor of interstitial fibrosis in the right atrial appendage (RAA) found in patients undergoing cardiac surgery [18]. Elevated galectin C levels have also been identified in patients who experienced recurrences of AF after catheter ablation [22,23].

Another biomarker associated with an increased risk of AF recurrence post-ablation is growth differentiation factor-15 (GDF-15), with a cut-off limit of 1287.3 ng/L [24].

Recently, the prognostic role of D-dimers has been emphasized, with elevated preoperative D-dimer levels being correlated with increased postoperative mortality in the medium and long-term follow-up. The inclusion of D-dimers in risk models can guide therapeutic management and improve the prognosis of these patients [25].

Plasma proteomic profiling is a new technique used by Lumisch, who identified a panel of 12 plasma proteins predictive of AF occurrence in patients with HCM. His study showed the alterations of the Ras-MAPK pathway, which are associated with an increased arrhythmic risk. The most important proteins in the panel are the following: prosapone (whose inactivity is associated with the impairment of the MAPK pathway), heat shock protein 70 (involved in sarcoplasmic calcium homeostasis, with its functional impairment being associated with an increased AF risk), kallikrein, the alpha receptor of immunoglobulin 2 (low concentration identifies a higher arrhythmic risk), as well as haptoglobin (a marker of hemolysis, correlates with increased gradients in the LVOT and a higher risk of AF). Additional studies on plasma proteomic profiling are required to validate this technique in patients with HCM. From a technical perspective, in addition to plasma proteins, evaluation of myocardial tissue fragments is also necessary [26].

Several studies have supported the prognostic value of identifying low-voltage areas during electrophysiological studies in patients with HCM and atrial myopathy [18]. Thus, the extent of low-voltage areas (>5% of the LA surface in sinus rhythm, >15% in AF, and voltage < 0.5 mV in sinus rhythm, <0.24 mV in AF), reveals an increased risk of recurrence after ablation [27].

In his study, Enriquez Vasquez demonstrated that patients with AF have electromechanical dissociation at the atrial level, which serves as an early indicator of atrial remodeling found in patients with newly onset AF. In these patients with recent onset of AF, atrial depolarization rates are faster than contractions recorded at this level. Atrial electromechanical dissociation has been shown to be a more accurate predictor of AF recurrence after catheter ablation than ultrasound parameters and blood biomarkers [28].

### 5.2. Multimodal Assessment of Atrial Myopathy and Thromboembolic Risk

Studies have emphasized the importance of assessing the structure and function of LA in patients with HOCM, regardless of their baseline rhythm, as venous stasis and atrial dysfunction activate Wirchow’s triad, increasing thromboembolic risk even in patients with a stable sinus rhythm [29,30].

Atrial myocardial involvement (fibrosis and hypocontractility) is associated with an increased risk of arrhythmogenesis, endothelial damage and secretion of prothrombotic factors (Il-6, il-8, and TNF-α). Studies have shown a close correlation with ischemic vascular accidents, including cryptogenic ones, regardless of the coexistence of AF. Atrial myopathy is a risk factor for the onset of AF [31].

Echocardiography, through classic parameters (LA antero-posterior diameter, LA maximal volumes measured by the Simpson biplane method, and LA ejection fraction), provides additional information about the risk of developing AF [32].

Furthermore, it has also been demonstrated that a decrease in LA global strain below 23.4% or reservoir strain below 20% increases the risk of AF, independent of the LA size [33].

A recent study in patients with HOCM suggests that atrial myopathy, assessed by LA reservoir strain, correlates with decreased exercise capacity and ventilatory efficiency, with cardiopulmonary exercise test demonstrating reduced peak VO2 and VE/VCO2 curves [34].

Evaluation of LA function through CMR has demonstrated the correlation between LA fibrosis and the risk of both new-onset AF and AF recurrence after radiofrequency ablation [35].

Additionally, a novel noninvasive CMR technique, the 4-dimensional flow technique, is superior to transesophageal echocardiography in assessing blood flow dynamics and identifying intra-atrial stasis and the risk of thrombosis [36].

Atrial myopathy is a generally accepted concept, correlated with thromboembolic risk among patients with HOCM, both in the presence and absence of AF. Atrial myopathy has been demonstrated using CMR, through findings such as atrial dilation, fibrosis and impaired mechanical function, all of which correlate with increased arrhythmic risk and decreased exercise capacity. Most of the studies are observational, and, consequently, large, prospective and randomized trials are needed to provide clinical validation. The indications for anticoagulation among patients with atrial cardiomyopathy, in whom AF has not been documented, remain under debate.

### 5.3. LA Function Assessed by Speckle-Tracking Echocardiography in Patients with HCM

The LA strain is the most relevant parameter of atrial function, comprising three phases:

- Reservoir phase: Begins at the end of ventricular diastole (mitral valve closure) and lasts until the mitral valve opens. It is represented by 3 components: the isovolumic contraction time, the ejection time and the isovolumic relaxation time. The atria fill with blood from the pulmonary veins or vena cava during this phase.
- Conduit phase: starts with the opening of the mitral valve, includes the diastasis period and lasts until the beginning of atrial contraction in patients with sinus rhythm.
- Contraction phase: this phase begins with the start of LA contraction and continues until the end of ventricular diastole (mitral valve closure) in patients with sinus rhythm [37].

Impairment of the LA reservoir strain is associated with atrial fibrosis identified on CMR and predicts the risk of new-onset AF [38].

Additionally, the LA dyssynchrony reflects the presence of atrial fibrosis and electrophysiological changes in the atrial wall, which predispose not only to the onset of new AF but also to the risk of recurrence after radiofrequency ablation [39].

The LA stiffness index (the ratio between E/E′ and peak atrial longitudinal strain) has been shown to be superior to global longitudinal strain and indexed LA volume in predicting low voltage areas [40].

AS dilation is also associated with AF in the perioperative period and with higher degrees of fibrosis [41].

Remodeling of gap junctions, the impairment of connexins and intra-atrial conduction delay are directly involved in atrial remodeling. Gap junctions are composed of connexins 40 and 43, and their impairment is associated with atrial dyssynchrony, as evidenced by atrial deformation [42].

Oxidative stress and elevated levels of reactive oxygen species lead to intracellular calcium overload. Reduced activity of sarcolemmal calcium channels is the main cause of impaired excitation–contraction coupling, being correlated to peak atrial strain decrease [43].

In conclusion, echocardiography is a useful tool for assessing atrial function and detecting early changes suggestive of an increased risk of new-onset AF.

### 5.4. Right Atrial Function Assessed by Speckle-Tracking Echocardiography in Patients with HCM

In patients with HOCM with preserved systolic function, echocardiographic evaluation revealed a predominant reduction in the LA reservoir and conduction strain, in contrast to the pump function, which was not affected [44].

LA dilation and contractile dysfunction assessed by speckle-tracking echocardiography are associated with the onset of perioperative AF [45].

RA strain can provide diagnostic and prognostic information in patients suspected of HCM, revealing early myocardial dysfunction.

In patients with more advanced stages of the disease, serial measurements of RA strain and strain rate are recommended, assessing contractile dysfunction and the severity of atrial stiffness, which has clinical and therapeutic consequences [44].

**Figure 1:**
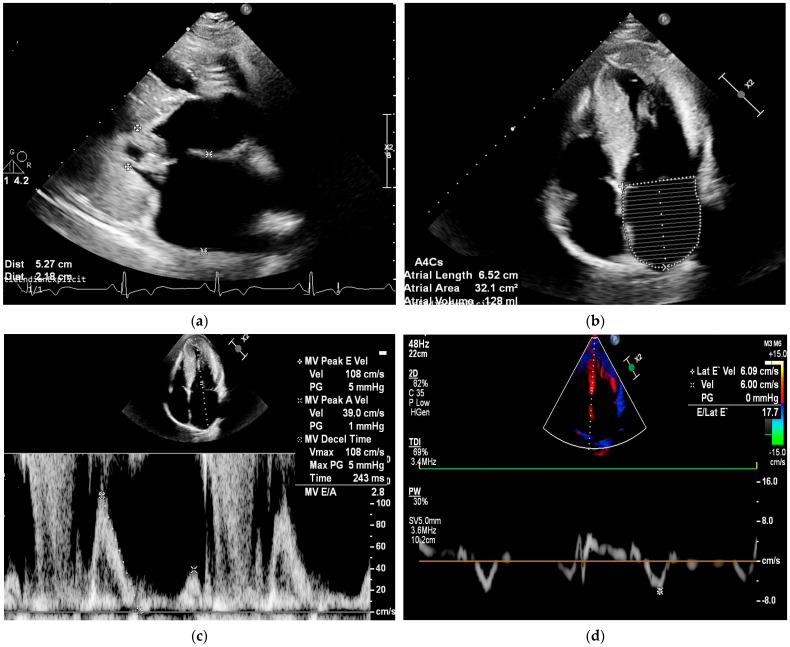

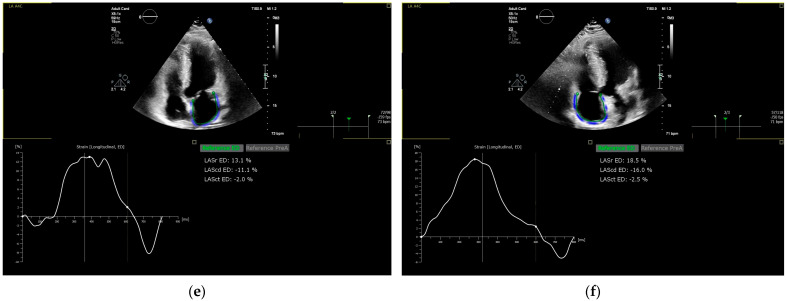
Clinical case of a patient diagnosed with HOCM, who experienced paroxysmal AF episodes, showing left and right atrial remodeling and elevated filling pressures, both predisposing factors to AF development. The images present echocardiographic measurements associated with AF onset in patients with HOCM. Images (**a**–**f**) were obtained from the institutional database and were anonymized. (**a**) Increased LA antero-posterior diameter (≥45 mm). (**b**). Marked dilation of the LA area (≥28 cm^2^) and volume. (**c**,**d**). Elevated left ventricular filling pressures (E/e′ ratio = 17.7). (**e**,**f**). Alterations of both left and right atrial strain.

In conclusion, speckle-tracking echocardiography provides important data on the severity of atrial myopathy, serving as a valuable tool for monitoring and adjusting therapeutic approaches in patients with HCM. Figure 1 summarizes some of the main echocardiographic data highlighting the severity of cardiac involvement in a patient with HOCM.

## 6. Treatment of AF in HCM

### 6.1. Pharmacological Treatment

Maintaining sinus rhythm is a particularly important therapeutic target, as the onset of AF in patients with HOCM significantly increases the risk of thromboembolic events, worsens heart failure symptoms, and leads to hemodynamic deterioration and unfavorable prognosis among these patients.

Amiodarone is a medication with a good safety profile, reducing the arrhythmic burden among these patients. Prolongation of the QT interval is rarely seen, and the proarrhythmic risk is also low. However, long-term use is associated with hepatotoxicity, thyroid dysfunction, and pulmonary toxicity, requiring discontinuation over time [42]. Studies have shown that after a period of discontinuation, the drug can be reintroduced in lower doses and is well tolerated. Treatment with amiodarone significantly reduced episodes of paroxysmal AF or supraventricular tachycardia, but with drug reactions.

Sotalol is the preferred antiarrhythmic in younger patients, which has been shown to reduce the frequency of atrial arrhythmic episodes and, consequently, AF episodes, increase exercise tolerance, and prevent ventricular tachycardia episodes. During treatment, monitoring the QT interval, renal function, serum potassium, and magnesium is recommended. In rare cases, sotalol may cause bronchospasm.

Beta-blockers increase exercise capacity, lower heart rate, reduce the severity of intraventricular obstruction, and reduce myocardial oxygen consumption. They have a class IA recommendation for pharmacological treatment in patients with symptomatic HOCM [46].

In September 2024, Kim Morris published a retrospective study that evaluated the postoperative evolution of 3532 patients who underwent septal myectomy between 2016 and 2021, with an average follow-up of 2.2 years per patient.

The study found an increased risk of AF or atrial flutter among patients with septal myectomy who received postoperative beta-blockers. Given the widespread postoperative use of beta-blockers in patients with HOCM, further studies are warranted to clarify this association [47].

Studies showed that calcium channel blockers (e.g., verapamil) improved LV relaxation and diastolic function and increased isovolumic relaxation time. However, some studies indicate that intravenous verapamil does not improve ventricular relaxation and may worsen hemodynamic parameters.

The 2023 ESC guidelines recommend a class I indication, verapamil and diltiazem (titrated to the highest tolerated doses), for symptomatic patients with HOCM who cannot tolerate or have contraindications to beta-blockers [48].

Disopyramide reduces the dynamic gradient in the outflow tract and LV contractility and reduces the anterior systolic movement of the mitral valve, maintaining the LV stroke volume preserved in patients with HOCM [49].

However, its antiarrhythmic effect is relatively low (35% after 3 years), and tachyphylaxis further reduces its effect. Patients may experience intolerance, given the anticholinergic effect. Additionally, given the QT prolongation effect, disopyramide increases the risk of ventricular tachycardia [50].

Class IC antiarrhythmics (flecainide and propafenone) are widely used in the general population for AF prevention, with a good safety profile, ensuring optimal rhythm control and reducing the frequency of ventricular arrhythmic episodes, without increasing cardiac mortality, in patients with nonischemic cardiomyopathy [51]. However, a study published by Garcia-Granja in 2017 suggested the proarrhythmic risk induced by the class I antiarrhythmics (flecainide and propafenone) use in patients with HCM, as these drugs prolong the QT interval, potentially predisposing to ventricular arrhythmias [52].

### 6.2. Comparison Between American and European Guidelines for AF Management

The management of AF in patients diagnosed with HCM has been presented both in the European Society of Cardiology (ESC) Guideline and the American Heart Association/American College of Cardiology (AHA/ACC) guideline, with notable convergence in recommendations, but some differences in conceptualization.

The main consensus between the ESC AF guideline and AHA/ACC recommendations is that HCM predisposes to a high risk of thromboembolism. Both guidelines recommend lifelong anticoagulation, independently of the CHA2DS2-VASC risk score, in all patients diagnosed with AF and HCM.

Direct oral anticoagulants (DOAC) are preferred (first-line option) over vitamin K antagonists (second-line option) in both European and American guidelines, unless they are contraindicated (class 1 recommendation).

In patients with HCM and AF who have contraindication to anticoagulation, LA appendage closure represents an option, as it is mentioned in the ESC (2020) guideline (class IIb). Based on patients’ characteristics, selection between Amplatzer and Watchmann occluders is individualized, being a safe procedure in patients with contraindication to long-term anticoagulation and high embolic risk [13].

The rhythm control strategy is another important point of the guidelines, because atrial contraction loss in AF determines worsening of heart failure. Both guidelines conclude that loss of atrial systole impairs ventricular filling, and, consequently, the rhythm control strategy is preferred compared to the general population. The ESC guideline supports an early rhythm control strategy, preventing atrial remodeling. The AHA/ACC guideline also acknowledges the benefits of an early rhythm control strategy (class 2a recommendation in both guidelines).

Regarding the antiarrhythmic drug therapy, the ESC guideline adopts a safety strategy, and there is no specific HCM approach. AHA/ACC guidelines favor amiodarone in HOCM; disopiramide has been prescribed for LVOT obstruction reduction, but its efficacy in AF is not well-established. The American guideline discourages the Class IC antiarrhythmic drugs (flecanide and propafenone), which should be used in the presence of an ICD.

Catheter ablation of AF has low efficacy, high recurrence and frequent need for repeated procedures in patients with HCM. Patient selection is crucial, as outcomes of radiofrequency ablation in HCM are inferior to those in patients without structural heart disease. American guidelines recommend catheter ablation as class 2a in these patients.

The differences between ESC and AHA/ACC guidelines are due to their approach: the ESC guidelines address these pathologies separately, whereas the American guideline integrates recommendations for both conditions in a single document, offering more integrated recommendations in this clinical scenario.

Despite differences in structure, these guidelines converge on main therapeutic principles, particularly initiation of anticoagulation regardless of the CHA2DS2-VASC score and the importance of the rhythm control strategy [53,54,55,56,57].

### 6.3. Non-Pharmacological Treatment of AF in HCM

A study involving 5161 patients divided into two groups (a group with AF and HCM and a control group with AF without HCM) concluded that patients with HCM presented larger atrial volumes, higher atrial filling pressures and less favorable reverse remodeling at 1 year, compared to those without HCM. In patients with a non-apical form of HCM, the rate of AF recurrences was significantly higher compared to those with an apical form of HCM. Additionally, the assessment of LA strain revealed that the non-apical HCM is associated with lower atrial strain, demonstrating more extensive myocardial fibrosis, more severe diastolic dysfunction and higher filling pressures compared to the apical form of HCM [58].

Septal myectomy in patients diagnosed with HOCM reverses atrial remodeling, leading to a decrease in mitral regurgitation severity and a reduction in LVOT obstruction.

Currently, myosin inhibitors are being intensively studied and have shown similar benefits compared to surgery, but their effect on atrial myopathy is still controversial, with a potential negative inotropic effect [4].

#### 6.3.1. Radiofrequency Ablation of AF in Patients with HCOM

Radiofrequency ablation plays an important role in reducing the frequency of AF recurrences among patients with HOCM, especially when antiarrhythmic medications are ineffective or poorly tolerated. Cohort studies and meta-analyses conducted so far have concluded that although transcatheter ablation reduces the frequency of AF recurrences in other patients, in patients diagnosed with HOCM, it is associated with a high recurrence rate, being less effective. Thus, in patients with HCM and radiofrequency ablation, 50% are free of AF episodes in the first year and 35% at 3 years, compared with 75% and 50%, respectively, in patients without HCM undergoing catheter ablation [59].

With repeated radiofrequency ablation procedures associated with high doses of antiarrhythmic treatment in the same patient, up to 70% of patients with HCM remain in sinus rhythm 5 years after the first ablation [60].

Regarding the ablation technique, some centers perform only pulmonary vein isolation, while other centers perform extensive lesions at the level of the left atrial posterior wall, in areas where fractioned electrograms with low voltage are identified.

The group led by Professor Efremidis demonstrated that patients with AF and HCM have significantly larger areas of atrial fibrosis compared to patients without HCM and that an extension > 14% with voltage < 0.25 mV is predictive of AF recurrence post-ablation, with a sensitivity and specificity of 100% [4].

Despite extensive ablation procedures, the rate of AF recurrence remains high among patients with HOCM, as proarrhythmic foci have also been identified in the coronary sinus, inter-atrial septum, right atrium (RA), or crista terminalis [61].

The success rate of AF ablation in HCM also varies depending on the timing of the procedure: it is more effective in younger patients, in less symptomatic patients, with less severe atrial remodeling, and in patients with paroxysmal AF [4].

Catheter ablation of AF carries low procedural risks (5% according to current studies); vascular complications at the access site are the most common. Major complications are rare (<1%) and are represented by tamponade or ischemic events. The complication rate is highly dependent on the expertise level, being recommended to refer patients to centers with high expertise and large volumes of procedures [62].

Regarding ablation techniques, no significant differences have been identified in recurrence rates between radiofrequency ablation and cryoablation [62].

Cryoablation of AF in patients with HOCM has low procedural risks and should be considered an effective rhythm control strategy. Studies demonstrated that almost 50% of patients experience no recurrence of atrial tachyarrhythmic episodes within 2 years post-cryoablation [63].

Table 1 summarizes selected studies evaluating AF ablation in HCM patients, including radiofrequency ablation, cryoablation, and pulsed field ablation, and provides a comparative overview of the outcomes and the short- to mid-term recurrence rate in these patients.

#### 6.3.2. Pulsed Field Ablation

Pulsed field ablation (PFA) is a new technique that creates non-thermal cardiac lesions through the mechanism of electroporation. Myocardial cells are exposed to high electric field variations, gaining increased permeability and being destroyed, without protein denaturation. Lesions are localized at the site of contact, without affecting surrounding tissues. Nerve and esophageal cells are more resistant to this procedure.

The editorial of Samy Gribissa enrolled patients undergoing both radiofrequency ablation and pulsed field ablation between 2016 and 2024. PFA was associated with a statistically significant lower rate of paroxysmal AF recurrence at 12 months of follow-up, compared to thermal ablation. Additionally, this editorial suggests shorter procedural times and lower incidences of heart failure compared to alternative procedures. Also, extra-PV radiofrequency ablation was associated with higher atrial arrhythmias recurrences. Preliminary data show a reduced impact of PFA on the contractile function of the left atrium and possible reduced atrial fibrosis compared to radiofrequency ablation. Additional data from large, randomized studies, carried out over a longer period of time, are needed to validate the results of this technique.

Another retrospective study, which compared two cohorts undergoing PFA versus thermal ablation, demonstrated the absence of atrial arrhythmic recurrence in 88% of individuals undergoing PFA, compared to 57% in the radiofrequency group. Preliminary data indicate the possibility of a favorable effect among patients with HCM undergoing PFA.

In conclusion, AF ablation, including new techniques such as PFA, represents therapeutic options in certain categories of patients with HCM. Success rate is influenced by the severity of underlying atrial disease, and repetition of these ablation procedures is frequently necessary. Multicenter prospective studies are needed to optimize ablation procedures and establish their long-term efficacy [64,65].

**Table 1:** Summarizes the main characteristics of the presented studies on AF ablation in patients with HCM.

Study	Year	Type of Study	Population	Sample Size	Type of Ablation	Recurrence/Follow Up	Key Notes
Castagno. et al. [60]	2021	Observational	HCM	116	PVI+/−additional lesions	High recurrence in persistent AF; acceptable rhythm control	Left atrial remodeling determines ablation success
Creta et al. [62]	2021	Multicenter Observational	HCM	137	PVI+/−additional lesions	Recurrence influenced by LA size and AF type Long time	Rate of recurrence determined by AF type and left atrial enlargement
Kim et al. [63]	2025	Registry	HCM	59	Cryoablation	Moderate success rates Mid-term	Succes influenced by atrial substrate and AF duration
Urbani et al. [65]	2024–2025	Observational	HCM	60	Pulsed-field ablation	PFA-safety profile, efficacy; data limited Short-mid term	PFA—potential advantage in reducing collateral tissue injury due to non-thermal mechanism. Comparable efficacy to thermal ablation

#### 6.3.3. Surgical Ablation of AF in Patients with HOCM

It involves removing the obstruction from the LVOT through septal myectomy and surgical isolation of the pulmonary veins or the Cox-Maze IV biatrial procedure. It does not further increase the operative risk, although it involves multiple incisions at the atrial level. Scientific data support a lower rate of AF episodes after surgical ablation, but a direct comparison between surgical and interventional techniques is difficult, given that the surgical procedure also involves the simultaneous performance of myectomy, which normalizes left intracavitary pressures [66].

Additionally, the surgical intervention involves the LA appendage excision, thus eliminating a potential source of thrombosis [67].

#### 6.3.4. Hybrid Ablation of AF

Minimally invasive surgical ablation in the atrial epicardial sites, combined with transcatheter endocardial ablation, along with LA appendage excision, is an aggressive technique sometimes used in patients with persistent AF without HCM. There are no studies yet evaluating the results of hybrid ablation versus transcatheter ablation in patients with HCM [68].

## 7. Treatment in HOCM

### 7.1. Pharmacological Treatment in HOCM

#### 7.1.1. Myosin Inhibitors

Mavacamten is a selective and reversible inhibitor of cardiac myosin, which alters the sarcomeric interaction between actin and myosin and decreases contractility. In patients with HOCM, it reduces dynamic obstruction in the LVOT, reduces myocardial stiffness and lowers intraventricular filling pressures. Its efficacy has been confirmed in 3 clinical trials: PIONEER-HCM, EXPLORER-HCM and VALOR-HCM. Favorable results were obtained in reducing the LVOT gradient both post-exercise and at rest, improving dyspnea scores, NYHA functional class, and Kansas score, increasing exercise capacity and improving myocardial oxygen consumption. Substudies of the EXPLORER-HCM study have demonstrated favorable effects in terms of reducing ventricular wall thickness, indexed LA volumes, and anterior systolic movement of the mitral valve, increasing septal e′ and reducing the e/e′ ratio.

Mavacamten received FDA approval in the USA in April 2022 and European Commission approval in June 2023. The initial dose is 2.5–5 mg, with progressive titration to a maximum dose of 15 mg/day, with patients being monitored under the CAMZYOS REMSLA Program every 12 weeks, or 4 weeks after the last dose adjustment. There have been cases of reduced LV ejection fraction < 50% or cases with AF onset, although these events were not directly linked to the medication. Drug interactions occur due to its metabolism via the cytochrome P450 pathway and potentiation of the negative inotropic effect in combination with beta-blockers or calcium-blockers. Additionally, it should not be administered to pregnant or breastfeeding women or to children [69].

In terms of subclinical myocardial dysfunction, the VALOR-HCM study demonstrated the most significant improvement in global LV strain in the first 32 weeks of mavacamten treatment, with favorable results observed up to week 56. Regarding the regional LV function, the most significant changes were observed in the antero-septal wall, where the most significant degree of hypertrophy was also found. A subgroup of patients had to discontinue mavacamten administration due to worsening of global longitudinal left ventricular strain (GLS). In these patients, lower baseline GLS values were found at the time of enrollment, compared to the rest of the study population.

Regarding right heart function, volumes, right ventricular free wall velocity, and TAPSE (tricuspid annular plane systolic excursion), all these parameters remained within normal limits despite myosin inhibitor administration. Regarding RV GLS, no notable changes were recorded during mavacamten treatment, with baseline values being lower than in normal individuals.

Studies are still ongoing to identify a GLS cut-off for the LV that can help determine the subgroup of patients with an unfavorable response to mavacamten [70].

Treatment with myosin inhibitors reverses LA remodeling, improving diastolic function and the degree of mitral regurgitation, similar to the effects observed after surgical myectomy. In contrast to myectomy, myosin inhibitors have a negative effect on atrial inotropism, as shown in preliminary small cohort studies, demonstrating a significant reduction in LA strain. It is still uncertain whether the effect of reversing LA remodeling or the reduction in atrial inotropism predominates in determining the risk of AF [4].

It is worth noting that the use of mavacamten has also been extended to patients who have previously undergone septal reduction therapies or aortic valve replacement, and who subsequently present with heart failure symptoms and recurrent LVOT obstruction. A study published by Daniele Massera in the Journal of the American Heart Association in 2025 enrolled 115 patients diagnosed with HOCM who had previously undergone septal reduction interventions (surgical myectomy or alcohol septal ablation) or ventricular pacing with a shortened atrio-ventricular interval, but who remained severely symptomatic. Most patients experienced symptomatic improvement, with a decrease in NYHA functional class of at least 1 grade, after Mavacamten initiation. However, 36% of the patients enrolled in the study did not respond to Mavacamten therapy, either due to persistent symptoms and persistent NYHA class (associated with dynamic gradient on Valsalva maneuver ≥ 30 mmHg, persistent diastolic dysfunction, signs of microvascular ischemia or deconditioning related to obesity) or required discontinuation of treatment due to adverse reactions (fatigue, dizziness, and LV dysfunction with LVEF < 50% on echocardiographic evaluation). In conclusion, this study demonstrated the effectiveness of mavacamten in the majority of patients with symptomatic HOCM and persistent severe obstruction despite prior septal reduction therapies [71,72].

Aficamten has a shorter half-life compared to mavacamten (3–4 days versus 7–9 days), which allows an easier dose adjustment. In phase-2 studies, it has demonstrated efficacy in reducing intraventricular gradient, improving NYHA class (for patients classified as NYHA class III), without any major adverse events [73].

Results from the FOREST-HCM trial with Aficamten over 48 weeks showed that nearly all patients who were eligible for septal reduction therapy at baseline were no longer eligible at the 6-month assessment and remained ineligible at 48 weeks after the initiation of Aficamten treatment. Additionally, only a small proportion of patients (4.3%) experienced a reduction in LV ejection fraction below 50%, which was reversible after treatment discontinuation, without worsening heart failure or AF occurrence during follow-up [74].

The MAPLE-HCM study demonstrated the superiority of Aficamten compared to metoprolol, with patients receiving Aficamten experiencing reduced LVOT gradients and LA volumes, and improved LV diastolic function, SAM, and mitral regurgitation compared to metoprolol. A modest reduction in global longitudinal strain, radial strain, and LV ejection fraction was also observed in the Aficamten group [75].

The OLE FOR–EST-HCM study followed up on 173 patients initially enrolled in REDWOOD-HCM or SEQUOIA-HCM for up to 48 weeks. Among these, 14 patients with new-onset AF or recurrent AF did not experience worsening heart failure, suggesting the safety of Aficamten in patients with AF. Overall, the OLE study underscores the safety, efficacy, as well as reduced incidence of newly diagnosed AF episodes with prolonged treatment with Aficamten [76].

#### 7.1.2. Cardiac Mitotropic Agents

Ninerafaxstat is a mitotropic agent that targets mitochondrial metabolism by inhibiting enzyme 3-ketoacyl CoA thiolase (3-KAT) (the last enzyme in the beta-oxidation pathway of fatty acids). This metabolic shift redirects myocardial energy utilization from fatty acid oxidation to glucose oxidation. Thus, the energy required for the oxidation of fatty acids is redirected for the oxidation of glucose, which consumes less energy for each molecule of ATP generated. Ninerafaxstat reduces oxidative stress and oxygen reactive species (ROS), which are known to be increased in patients with HCM. Compared to myosin inhibitors, ninerafaxstat improves the exercise capacity in patients with non-obstructive HCM, and from preliminary studies, it does not seem to affect LVEF. Patients with mutations in the MYBPC3 and MYH7 genes show more cardiovascular involvement and seem to benefit from treatment with myosin inhibitors. Patients with severe symptoms (NYHA class II–III) and those with non-obstructive HCM are more suitable for metabolic modulators, with ninerafaxstat being the most appropriate option.

Further studies are needed to establish the long-term safety and efficacy of these new-generation agents in various patient groups [77].

### 7.2. Non-Pharmacological Treatment in HOCM

#### 7.2.1. Septal Myectomy Versus Alcohol Septal Ablation in Patients with HOCM

A meta-analysis published by Karla Inestroza and Ivan Mijares-Rojas in 2024 concludes that an individualized approach is optimal, depending on the pathology and comorbidities of each patient. The analysis, which included 15,119 patients treated between 2011 and 2019, highlights the advantages and disadvantages of each technique. Patients undergoing septal myectomy have higher in-hospital mortality and a higher risk of periprocedural complications (postprocedural ischemic stroke, acute renal failure, vascular complications, ventricular septal defect, cardiogenic shock, sepsis, and major conduction block), while patients undergoing alcohol septal ablation (ASA) have a higher risk of third-degree AV block requiring permanent pacemaker, major right bundle branch block, both ventricular and supraventricular tachyarrhythmias, higher residual LVOT gradients, and a higher need for repeat septal reduction therapy (SRT) [78].

Septal myectomy, rather than ASA, is recommended in children with an indication for SRT, as well as in adults with an indication for SRT and other lesions requiring surgical intervention (e.g., mitral valve abnormalities, which are frequent in HOCM) [79].

At the same time, the patient’s preference must first be considered, as it will guide therapeutic management.

#### 7.2.2. Percutaneous Septal Radiofrequency Ablation in Patients with HOCM

This is an effective and safe technique, with patients undergoing this procedure having a favorable outcome at 6 months of follow-up. Significant reduction in LVOT gradients was observed, both at rest and during exertion, as well as a reduction in interventricular septal thickness, an improvement in diastolic function, and an increase in the distance walked during the 6 min walk test. None of the patients who underwent percutaneous septal radiofrequency ablation developed complete atrioventricular block and bundle branch block post-intervention. In conclusion, percutaneous septal ablation seems to be a safe and effective procedure.

Bipolar percutaneous endocardial septal radiofrequency ablation is a novel technique, published by Aiju Tian in 2025 in JACC. This approach involves a dual percutaneous access, both at the right and the left ventricles. By performing the anatomical map of both ventricles, the septal region involved in the obstruction is precisely defined, as well as the location of the bundle of His, the branches and the Purkinje network.

Following ablation, there was a reduction in the LVOT gradient (nonsignificant in the isoproterenol testing performed postintervention) and an improvement in NYHA functional class and Kansas City Cardiomyopathy Questionnaire. Bipolar percutaneous radiofrequency ablation is a safe and rapid procedure, with no major complications, preserving the cardiac conduction system. Further studies are needed to validate the procedure, with long-term follow-up and the definition of optimal ablation parameters to create effective, controlled lesions [80].

#### 7.2.3. Surgical Treatment in HOCM

Septal myectomy was first performed in 1961 by Morrow and involved the resection of a significant segment of the interventricular septum through a transaortic approach. It remains the standard treatment for patients who remain symptomatic despite maximal medical therapy [81,82].

The phenotype with subaortic septal obstruction represents the most frequent form of HOCM, alongside less frequent cases with midventricular or apical obstruction, which can be isolated or associated with the form involving subaortic obstruction.

In most HCM centers, transaortic septal myectomy is the most commonly used surgical intervention for symptomatic patients with HOCM who are refractory to maximal medical therapy [83].

The mortality associated with septal myectomy has progressively decreased over the years, currently reaching <1% in experienced centers, with an operative success rate of 90–95%. This success rate is also influenced by the need for concomitant procedures that increase the operative risk (for example, concomitant coronary artery bypass surgery) [84].

The retrospective study published in 2024 by Ivana Seia, which analyzes the effect of extensive septal myectomy, highlights a significant improvement in the NYHA functional class of the patients, with 89% being asymptomatic in the long term. Regarding the echocardiographic evaluation, there was significant improvement in diastolic function after the removal of dynamic obstruction, a reduction in the severity of mitral regurgitation, lowered intraventricular filling pressures, reverse remodeling of the LA, as well as decreased pressures in the pulmonary circulation. However, a decrease in the LV ejection fraction was recorded, which was explained by both the onset of left bundle branch block postmyectomy leading to intraventricular dyssynchrony and by the effect of extracorporeal circulation on the myocardium. The authors report a series of complications associated with extensive septal myectomy: all patients developed postoperative major left bundle branch block, 7.4% required permanent pacemaker due to the onset of postoperative complete atrio-ventricular block, 3.1% had ventricular septal defect, perioperative mortality was 1.06%, and long-term mortality was 9.5% (including 3.2% deaths due to other comorbidities) [85].

Evaluation of patients who have previously undergone septal myectomy using stress echocardiography and cardiopulmonary exercise stress testing demonstrated, for the majority of them, significant improvement in exercise capacity, with an increase in the VE/VCO2 ratio, and improvement in peak VO2. However, approximately 26% of patients were nonresponders, predominantly patients with advanced preoperative HF, pre-existing AF and lack of participation in cardiac postoperative rehabilitation programs [86].

Regarding postoperative patient monitoring, it is important to evaluate latent LVOT obstruction by stress echocardiography, since the detection of positive criteria (exercise-induced hypotension or a dynamic LVOT gradient > 50 mmHg) predicts the future appearance of progressive heart failure symptoms, recurrence of LVOT obstruction, and the need for reintervention [4].

Conversely, left ventricular remodeling following septal myectomy continues beyond the postoperative period, as shown by a recent study that analyzed the CMR results at 6 months postoperatively. At this time point, regression of cellular hypertrophy and diffuse interstitial fibrosis, along with a reduction in left ventricular mass, was observed, while focal fibrosis remained largely unchanged [87].

In addition to the transaortic approach, scientific data also mention transmitral myectomy. This approach allows for simultaneous visualization of the mitral valve and interventricular septum, facilitating the treatment of mitral valve pathology. However, there are several limitations of this method, including: the intervention on the septum and mitral valve may be restricted due to reduced LA compliance and a small ventricular cavity, and there is a higher risk of damaging the conduction system and a significant risk of developing perioperative AF due to LA manipulation [88].

Septal myectomy performed using the two approaches mentioned above, under cardiopulmonary bypass, is associated with increased surgical risk and greater surgical trauma. Classic surgical techniques for resection of hypertrophied interventricular septum involve difficult control of the approach angle, the resection depth, and the precision of tissue removal. Transapical septal myectomy guided by transesophageal echocardiography is a new surgical technique that involves minimally invasive resection of the interventricular septum, representing a less traumatic alternative, as it is performed without cardiopulmonary bypass and allows the real-time evaluation of the surgical intervention. Compared to the modified Morrow procedure, transapical myectomy has the advantages of shorter operative times, avoidance of sternotomy, lower mortality rates, and improved control of septal resection [89].

The specialized literature further reports HCM cases presenting with midventricular obstruction or with both subaortic and midventricular obstruction, highlighting the importance of accurately identifying the midventricular involvement. The latter is confirmed on echocardiographic evaluation when the distance between the anterolateral papillar muscle and the interventricular septum is ≤8 mm. These patients are candidates for extended myectomy toward the left ventricular apex; otherwise, they are at risk of postoperative residual obstruction and may require surgical reintervention. Additionally, studies have shown that patients with mid-ventricular obstruction have a higher risk of developing LV apical aneurysm, refractory heart failure, higher NYHA functional class and elevated NT PRO-BNP levels [90].

Multimodal evaluation of the mitral valve in HOCM with LVOT obstruction using both transthoracic and transesophageal echocardiography, and also CMR, is recommended due to the coexistence of mitral valve apparatus abnormalities contributing to significant mitral regurgitation. Surgical intervention on the mitral valve should be considered in these cases. The cause of significant mitral regurgitation in patients with HOCM is the reduced coaptation of the mitral leaflets, the coaptation point being anteriorly displaced in the LVOT, resulting in a jet of mitral regurgitation directed towards the posterior wall of the LA [91].

In addition to anterior systolic displacement of the mitral valve, there are other causes of significant mitral regurgitation: dilatation and degenerative calcification of the mitral annulus, which may lead to a centrally oriented jet or multiple regurgitation jets.

Mitral valve replacement has been associated with poor outcomes in patients undergoing surgery for HOCM [92].

Mitral valve repair associated with septal myectomy consists of: plication of the anterior mitral leaflet, repositioning of the papillary muscles, and resection of the secondary chordae of the anterior mitral leaflet. The aim is to reduce obstruction and gradient in the left ventricular ejection tract, reduce the anterior mitral leaflet mobility, and shift the mitral coaptation point posteriorly [83].

As an alternative to the classic deep septal myectomy, a superficial (shallow) septal myectomy combined with resection of secondary chordae attached to the anterior mitral valve and mobilization of the papillary muscles has been described. This technique developed by Dorobanțu based on Ferrazzi’s approach allows for concomitant transaortic mitral valve repair, avoiding extensive septal myectomy, with the latter being associated with high morbidity in centers with less surgical experience [83].

Similar results come from a 2025 meta-analysis conducted in the Netherlands, which demonstrated that combined intervention resulted in an average postoperative gradient of 11 mmHg, decreased severity of mitral regurgitation and overall improved survival with 1-year mortality rates of only 0.6% [93].

The surgical technique combining septal myectomy with anterior mitral leaflet extension (introduced in 1986) did not show advantages over isolated septal myectomy, being time-consuming, increasing the risk of postoperative complications, and the need for permanent cardiac pacing and reintervention at 3 years, due to dehiscence of the pericardial patch. Consequently, this technique has been largely abandoned, as shown by the retrospective study published in 2025 by Tjin Heeringa [93].

**Figure 2:**
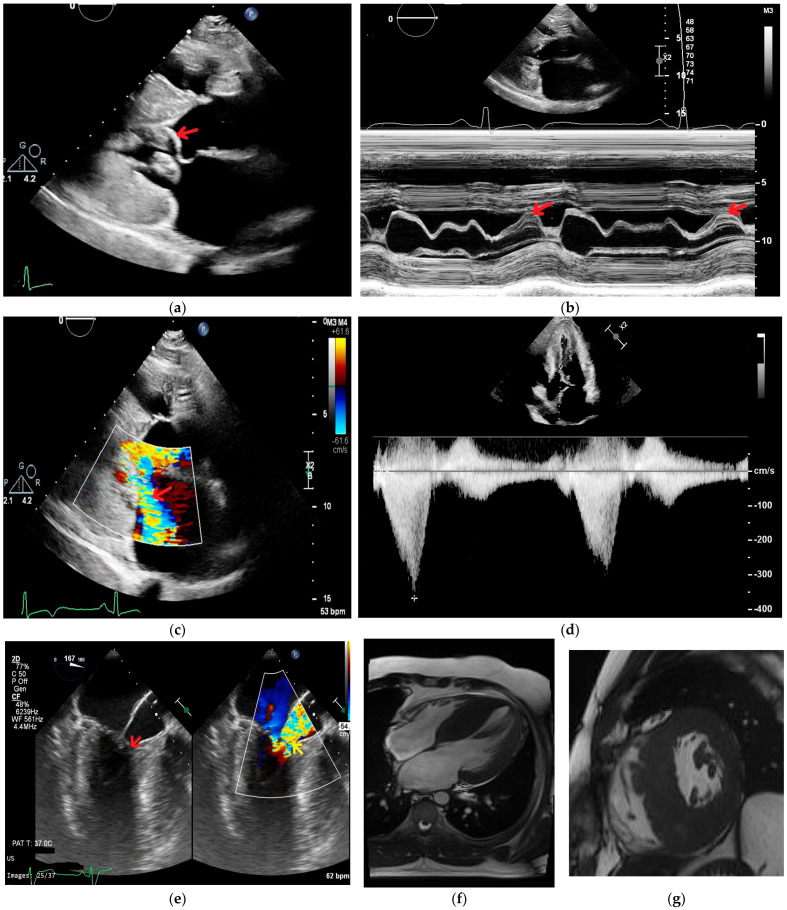

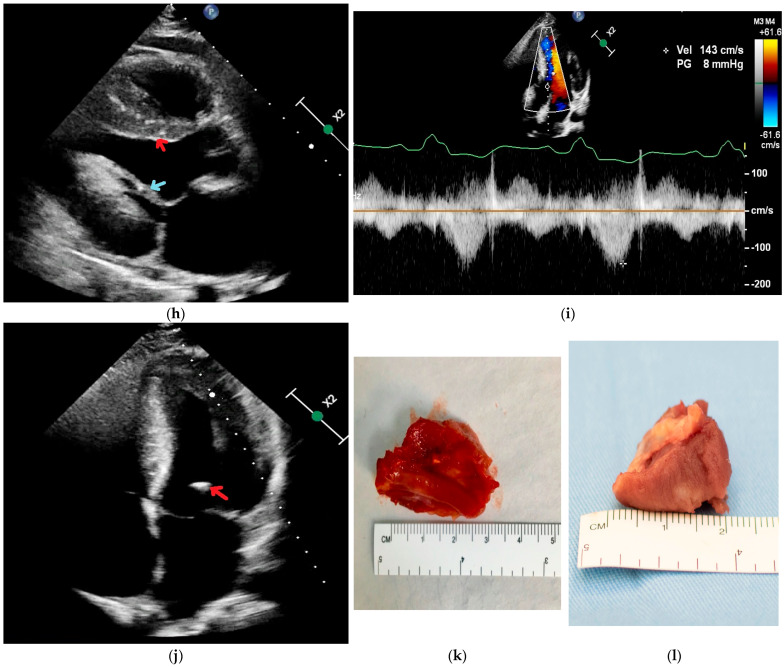
Clinical case of a patient diagnosed with HOCM who underwent myectomy and MV repair. Images (**a**–**k**) were obtained from the institutional database and were anonymized. (**a**) SAM of the MV (arrow), with significant obstruction in the LVOT. (**b**) M mode shows systolic anterior motion of the MV (arrow) and chordae. (**c**) Color Doppler image highlighting LVOT flow turbulence and moderate mitral regurgitation (arrow) secondary to SAM. (**d**) Severe LVOT obstruction secondary to SAM, with a peak gradient of 52 mmHg during the Valsalva maneuver. (**e**) Transesophageal echocardiography 3 C view showing LVOT obstruction (arrow). (**f**,**g**) CMR images demonstrate severe LV concentric hypertrophy. (**h**) Postoperative transthoracic echocardiography demonstrates resolution of SAM and LVOT obstruction; red arrow indicates septal myectomy, and blue arrow indicates anterior mitral leaflet plication. (**i**) Continuous wave Doppler shows a peak postoperative LVOT gradient of only 8 mmHg. (**j**) MV repair with plication of the anterior leaflet (arrow) results in loss of anterior movement of the MV. (**k**,**l**) Myectomy pieces performed at the anterior and posterior interventricular septum.

Figure 2 illustrates the pre- and postoperative echocardiographic images of a patient who underwent septal myectomy and MV repair.

## 8. Genetic Therapy

Genetic therapy represents a promising approach for patients diagnosed with HCM, aiming to correct the genetic mutations responsible for this pathology. Preclinical studies have demonstrated that tools such as adenine base editors and CRISP technology can correct the heterozygous mutation in carrier mice. Mutations in genes encoding the myosin heavy chain have been successfully corrected, preventing the appearance of hypertrophy, promoting left ventricular remodeling and reducing myocardial fibrosis. New methods of genetic treatment are being evaluated, including gene replacement, genome editing and modulation of key signaling pathways. However, the long-term efficacy of gene therapies is uncertain, since some of the newly introduced genes appear to have a limited duration of expression, potentially predisposing to relapse [94].

## 9. Discussions

The last 10 years have brought new insights and remarkable progress in the diagnosis, natural history and treatment of HCM. While, in the past, it was considered a disease with a poor prognosis, it is now considered compatible with a normal life expectancy, with potential complications during its progression being limited by multiple therapeutic options.

Studies that have analyzed patients with HCM are numerous, mostly observational or cohorts, and have identified a series of clinical, imaging and genetic parameters that would correlate with the onset of AF among patients with HCM. But, until now, there is no evidence to indicate the need for anticoagulation in patients with HCM without documentation of AF. The stratification of the AF onset risk by multiple variables is an extremely valuable tool for the clinical management of these patients, allowing for more careful monitoring and early detection of arrhythmias.

Atrial myopathy also proved to be a key determinant for thromboembolic risk in patients with HCM, extending beyond the presence of AF. Atrial remodeling and impaired atrial mechanical function promote blood stasis and thrombus formation even in the absence of AF.

Identification of patients at high thromboembolic risk will allow for an improvement in the prognosis and quality of life. In this regard, additional studies are needed to evaluate the benefit of prophylactic anticoagulant treatment in patients with HCM with a high risk of thrombosis and AF onset.

Invasive septal reduction treatment has brought major benefits in the management of symptomatic patients with HOCM. While its direct effect on AF is limited, relieving LVOT obstruction reduces atrial pressures and may improve rhythm control.

Both European and American guidelines recommend catheter ablation for symptomatic AF. Ablation outcomes vary depending on patient selection, degree of structural atrial remodeling, procedural technique and concomitant therapies. Transcatheter ablation is an important technique for restoring sinus rhythm in patients with HCM and atrial fibrillation. Studies conducted to date indicate a lower efficacy compared to patients without HCM. Introduction of the PFA procedure, as a new, non-thermal technique, suggests that it may be feasible in patients with HCM, possibly with a superior safety profile, minimizing damage to surrounding tissues.

Alongside surgical or interventional treatments, pharmacological treatment (especially myosin inhibitors such as mavacamten and aficamten) improves quality of life, minimizes morbidity and improves survival. Preliminary studies suggest potential effects in decreasing atrial stretch, which may influence AF onset risk.

Additionally, the mitotropic agent ninerafaxstat has demonstrated its efficacy in both obstructive and non-obstructive forms of hypertrophic cardiomyopathy in phase-2 clinical trials.

Although gene therapy is a promising approach, available studies are currently restricted to the preclinical phase.

## 10. Limitations

This manuscript is a narrative review and not a systematic one; therefore, it has inherent limitations. There are a number of studies selection bias, which may favor studies reporting positive results. Many studies included a small number of patients, reducing their statistical power. Also, the characteristics of the populations, such as age, sex, multiple risk factors, genetic variability and the complex interaction between them, complicate extrapolation to the HCM population.

Furthermore, most studies have short observation periods, whereas the natural course of HOCM and its complications requires long-term follow-up. Additionally, most of the cited studies are retrospective, which is also a source of bias, as disease progression and complications development may not be fully noted.

Large, multicenter, prospective studies are needed to provide clear evidence regarding AF and malignant ventricular arrhythmias risk in patients diagnosed with HOCM. Consequently, early implementation of personalized treatments will potentially improve survival and quality of life in patients diagnosed with HOCM.

## 11. Conclusions

HOCM is a genetic disease frequently encountered in the general population. Early identification of patients with HCM at high thromboembolic risk and those at risk of new-onset AF is particularly important, allowing optimal therapeutic management and the prevention of potentially catastrophic consequences.

## Data Availability

No new data were created or analyzed in this study.

## Abbreviations

The following abbreviations are used in this manuscript:

AF	Atrial Fibrillation
CMR	Cardiac Magnetic Resonance
GLS	Global Longitudinal Strain
CRISP	Clustered Regularly Interspaced Short Palindromic repeats
eGFR	Effective Glomerular Filtration Rate
HCM	Hypertrophic Cardiomyopathy
HOCM	Hypertrophic Obstructive Cardiomyopathy
IVS	Interventricular Septum
LA	Left Atrium
LAEF	Left Atrial Ejection Fraction
LV	Left Ventricle
LVOT	Left Ventricular Ejection Fraction
PFA	Pulsed Field ablation
RAA	Right Atrial Appendage
RV	Right Ventricle
SRT	Septal Reduction Therapy
